# The COVID Complex: A Review of Platelet Activation and Immune Complexes in COVID-19

**DOI:** 10.3389/fimmu.2022.807934

**Published:** 2022-03-14

**Authors:** Stefan D. Jevtic, Ishac Nazy

**Affiliations:** ^1^ Department of Medicine, McMaster University Medical Centre, McMaster University, Hamilton, ON, Canada; ^2^ McMaster Centre for Transfusion Research, McMaster University, Hamilton, ON, Canada

**Keywords:** COVID-19, platelet, antigen-antibody complex, immune complex, thrombosis, thrombocytopenia, heparin, VITT

## Abstract

Coronavirus disease 2019 (COVID-19) is a highly prothrombotic viral infection that primarily manifests as an acute respiratory syndrome. However, critically ill COVID-19 patients will often develop venous thromboembolism with associated increases in morbidity and mortality. The cause for this prothrombotic state is unclear but is likely related to platelet hyperactivation. In this review, we summarize the current evidence surrounding COVID-19 thrombosis and platelet hyperactivation. We highlight the fact that several studies have identified a soluble factor in COVID-19 patient plasma that is capable of altering platelet phenotype *in vitro*. Furthermore, this soluble factor appears to be an immune complex, which may be composed of COVID-19 Spike protein and related antibodies. We suggest that these Spike-specific immune complexes contribute to COVID-19 platelet activation and thrombosis in a manner similar to heparin-induced thrombocytopenia. Understanding this underlying pathobiology will be critical for advancement of future research and therapeutic options.

## Introduction

Coronavirus disease 2019 (COVID-19) is a respiratory infection caused by the severe acute respiratory syndrome coronavirus 2 (SARS-CoV-2) (1, 2). It has resulted in a global pandemic and is characterized by a highly inflammatory and prothrombotic state. Pulmonary involvement is the primary clinical manifestation but subsequent multi-organ failure and death can occur in severe cases (3–5). The prevalence of COVID-19 thromboembolism is quite variable across studies but appears to be highest in the critical care population, where estimates range from 20-65% (5–9). These can be both arterial and venous thromboses, making COVID-19 a unique prothrombotic state. Although the exact mechanisms underlying thrombosis are likely multifactorial, mounting evidence suggests that platelets play a crucial role.

Platelets have long been known to function as mediators of thrombosis and hemostasis but have only recently gained recognition in their role as immune mediators (10, 11). These anucleate cells mediate various immune related roles throughout the body, from antigen presentation to immune complex signaling. Of particular interest is their role in viral infection, where platelets are able to internalize and degrade pathogen as well as release soluble immune mediators (12). This likely contributes to their important role in COVID-19 and explains, at least in part, how platelet hyperactivation increases thrombotic risk.

In this review, we summarize the general thrombotic nature of COVID-19 and the importance of understanding this process in regards to therapeutic options. Subsequently, we review platelet physiology and their current recognized functions as immune cells. We also review platelet activation in heparin-induced thrombocytopenia (HIT) and how this parallels platelet activity in COVID-19. Finally, we discuss the novel entity of vaccine-induced thrombotic thrombocytopenia (VITT) and the role of platelets in this unique COVID-19 associated disorder.

## COVID-19 and Thrombosis

COVID-19 is a severe viral infection that was identified in Wuhan, China in late 2019. It initially presents as a respiratory tract infection, including fever, dyspnea, and myalgias, but can rapidly progress to a more severe form (1). These critically ill COVID-19 patients are characterized by excess inflammation and a prothrombotic state. COVID-19 thrombosis features both arterial and venous thromboembolic events, often with concomitant thrombocytopenia (7, 13). In one retrospective study of 1476 hospitalized COVID-19 patients, 20.7% were found to have thrombocytopenia (where 125 x 10^9^/L was the lower limit of normal) (14). The degree of thrombocytopenia has also been shown to independently associate with mortality outcomes, suggesting that platelet activation plays an important role in disease severity (14).

Thrombosis is particularly prevalent amongst critically ill COVID-19 patients, with some studies identifying deep vein thrombosis in up to 79% of patients through ultrasound screening (9). Unusual thrombi are also more prevalent amongst COVID-19 patients, including ischemic stroke, limb ischemia, and aortic thrombi (15). Up to 27.6% of thromboembolic events in critically ill patients occur even in the setting of prophylactic anticoagulation, emphasizing the extreme nature of this thrombotic state (16). Therapeutic dosing of anticoagulation may thus be required in COVID-19 hospitalized patients.

This observation has resulted in several randomized clinical trials demonstrating a benefit of therapeutic-dose low molecular weight heparin in hospitalized COVID-19 patients. In the ATTACC, ACTIV-4a, and REMAP-CAP multi-platform trial, therapeutic doses of heparin increased the probability of organ support-free days in non-critically ill patients (odds ratio 1.27, 95% credible interval 1.03-1.58) (17). However, there was no significant benefit for survival until hospital discharge, although there was a trend towards benefit (adjusted odds ratio 1.21, 95% credible interval 0.87-1.68). Interestingly, the RAPID trial did demonstrate a significant mortality benefit from therapeutic heparin in hospitalized, non-critically ill patients (odds ratio 0.22, 95% confidence interval 0.07-0.65) (18). Therefore, therapeutic anticoagulation is likely to benefit COVID-19 patients who are hospitalized without critical illness. It should be noted, however, that therapeutic anticoagulation showed no benefit to mortality or reduced organ support in critically ill COVID-19 patients (19). It may be that anticoagulation in these patients was introduced at an overly advanced stage of disease. Nonetheless, it implies that critically ill patients differ significantly in their underlying physiology and require unique therapies.

## Platelets and Their Role as Immune Cells

Prior to delving into platelet activation in COVID-19, it is important to gain a basic understanding of how platelet synthesis and function are intimately related to immunity. Platelets are produced in the bone marrow from progenitor cells, termed megakaryocytes, through a complex process of hematopoietic stem cell differentiation (20). Their production is primarily driven by the cytokine mediator, thrombopoietin (TPO), which is synthesized by both the liver and kidneys. TPO is known to be upregulated by inflammatory cytokines, such as IL-6, and contributes to the rapid platelet production seen with inflammation (21). This is secondary to a subgroup of “pre-differentiated” stem cells that are biased towards the megakaryocyte lineage and rapidly differentiate on TPO exposure (22–24). The hematopoietic system is thus efficiently designed to produce platelets in the context of infection, suggesting an important role in immunity.

Once released into circulation, platelets are equipped with various intracellular materials (over 300) to mediate their effector functions (25). These include inflammatory cytokines (e.g. IL-1β), procoagulant factors (tissue factor, serotonin), and angiostatic molecules (platelet-factor 4/PF4) (26–28). These molecules are released upon platelet activation, which is mediated through various cell surface receptors. Many of these cell surface receptors also contribute to immune cell interaction and function. For example, the GPIb receptor is normally involved in platelet adhesion at sites of vascular injury through von Willebrand factor binding. However, GPIb is also capable of binding to von Willebrand factor exposed on immune cells infected with bacterial pathogen, such as hepatic Kupffer cells (29). It has been shown in a mouse model that this interaction is crucial for platelet aggregation around infected cells and host survival. P-selectin is another platelet surface receptor that is known to be upregulated with platelet activation. It is capable of binding to leukocytes through the P-selectin glycoprotein ligand-1 to mediate intracellular leukocyte signaling and neutrophil rolling (30–32). This process is crucial for leukocyte mobilization and concentration at sites of infection. Indeed, the P-selectin dependent interaction between neutrophils and platelets has been shown to contribute to acute lung injury in mouse models (33, 34). Platelet depletion or P-selectin inhibition both reduced subsequent neutrophil recruitment and lung injury. Platelets are thus equipped, through both intracellular and cell surface proteins, to mediate various immune functions. These interactions may contribute to the lung pathology seen in COVID-19 through immune cell recruitment.

## Immune Complexes Are Capable of Platelet Activation

Immune complexes are important initial defenses against pathogen infection and are formed from antibody binding to soluble antigen. They often consist of immunoglobulins (Ig) of the IgG or IgM type but can also be IgA (35, 36). Immune complexes primarily mediate function through binding to cell surface receptors found on various cell types, including platelets. Most binding occurs through the Fcγ receptors II (FcγRII) and III, which are either activating (a) or inhibiting (b), respectively (35). Platelets contain only one Fc receptor (FcγRIIa) on their surface and thus are able to bind IgG-specific immune complexes (37). Immune complex binding to the platelet receptor leads to subsequent activation and release of intracellular molecules such as serotonin. This promotes a prothrombotic state and has been implicated in various autoimmune conditions. The most well-characterized platelet-mediated immune complex disorder is heparin-induced thrombocytopenia (HIT) (38, 39).

HIT is a prothrombotic autoimmune disorder characterized by the presence of thrombocytopenia (low platelets) and thrombosis that shares many features with COVID-19. It most commonly presents in hospitalized patients who are receiving unfractionated heparin anticoagulation and is characterized by antibodies targeting platelet factor 4 (PF4)-heparin complexes (39). PF4 is a positively charged protein released from platelets that is capable of binding negatively charged molecules, such as heparin (40). Certain individuals develop anti-PF4/heparin IgG antibodies that form immune complexes. These immune complexes activate the FcγRIIa on platelets resulting in thrombocytopenia that is often accompanied by thrombosis, which is secondary to the release of serotonin and other procoagulant platelet microparticles. Circulating anti-PF4/heparin antibodies can be found in up to 50% of patients exposed to heparin (41, 42). However, only a minority of these will be functional and lead to disease presentation. This is secondary to the unique epitope specificity required for immune complex formation and platelet activation (43). Therefore, it is important to use functional platelet activation assays to diagnose HIT.

**Figure 1:**
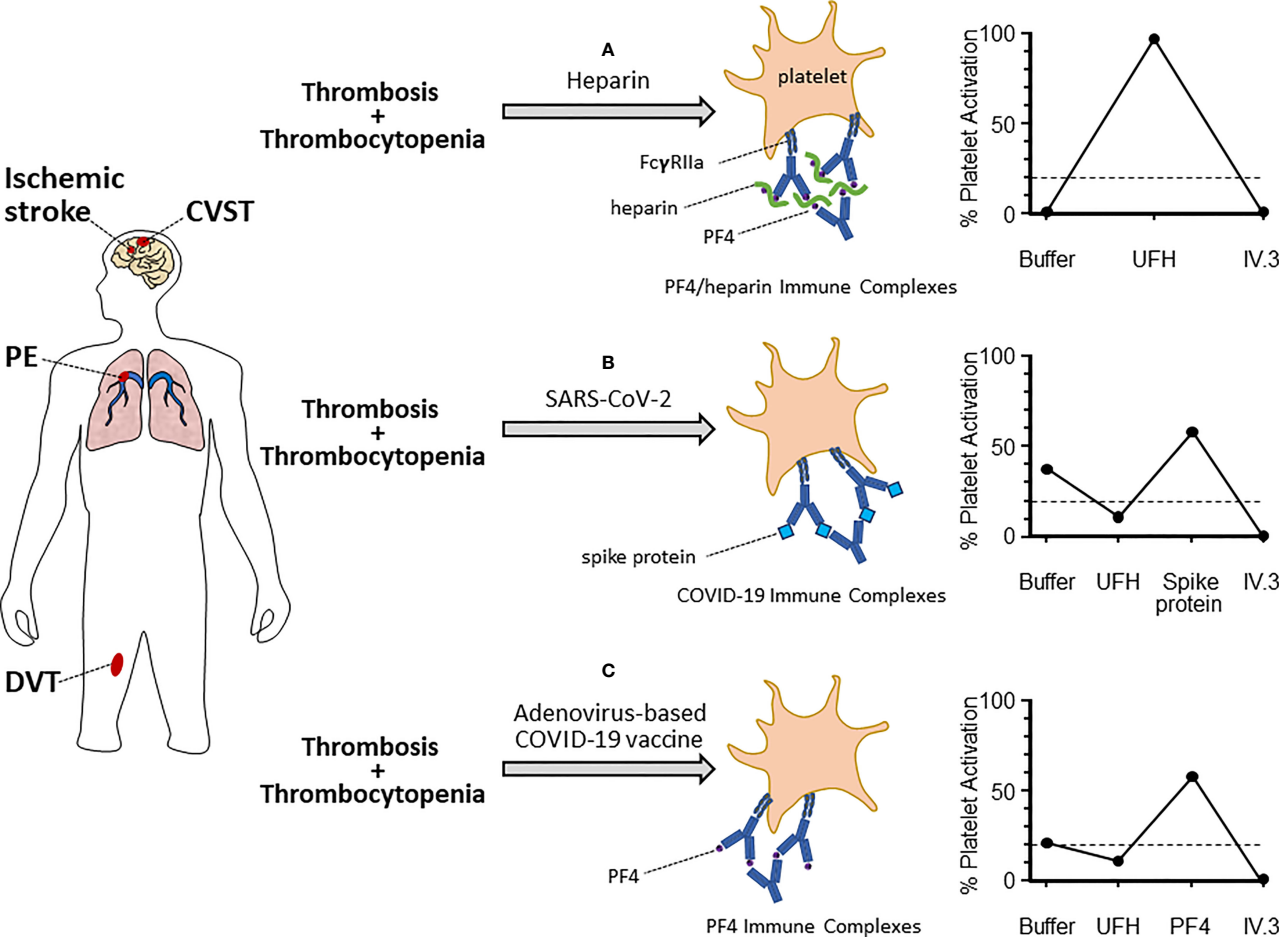
Platelet activation mechanisms and assay patterns in various thrombotic-thrombocytopenic syndromes. Patients who present with evidence of thrombosis (arterial or venous) and thrombocytopenia should be considered for hematology referral and specialized testing for platelet activation disorders. These can include heparin-induced thrombocytopenia (HIT, **A**), COVID-19-related platelet activation **(B)**, and vaccine-induced thrombotic thrombocytopenia (VITT, **C**). The suspected platelet activation disorder will depend on the clinical context and known exposure to antigen (e.g. heparin, SARS-CoV-2, or adenovirus-based COVID-19 vaccine). Each of these disorders is characterized by platelet activation through the FcγRIIa *via* unique immune complexes. These include: anti-PF4/heparin (HIT), unidentified immune complexes (COVID-19), or anti-PF4 (VITT). Serum testing from patients will also feature unique platelet activation schemas in functional activation assays, such as the serotonin release assay (examples shown on the right). In the classic HIT-SRA (top right), addition of exogenous UFH significantly increases immune complex formation and platelet activation, which is inhibited by IV.3. Contrastingly, UFH inhibits immune complex formation in COVID-19 and VITT thrombotic patients. Instead, alternate antigens (Spike protein and PF4, respectively) are required for significant platelet activation. Dashed lined represents 20% platelet activation, which is the positive cut-off for the SRA.

One of the international reference assays for diagnosing HIT, developed at our institution, is the serotonin release assay (SRA). Briefly, platelets from healthy donors are incubated with radioactive ^14^C-serotonin allowing uptake into platelets. These modified platelets are subsequently exposed to patient plasma, in the presence and absence of heparin, which allows formation of anti-PF4/heparin immune complexes (44). These immune complexes subsequently activate platelets through the FcγRIIa leading to release of ^14^C-serotonin, which is then measured by beta radioactivity. The addition of exogenous heparin is vital for this activation in HIT to facilitate formation of appropriate antigen complexes. However, certain samples tested in the SRA for HIT will demonstrate heparin-independent platelet activation (45). This is inconsistent with a diagnosis of classical HIT, meaning that the assay may detect additional mechanisms of platelet activation. Therefore, the SRA can be modified to study platelet hyperactivation in novel diseases, such as COVID-19 (see **Figure 1**).

## Platelets are Hyperactivated in Critically Ill COVID-19 Patients

To this point, many studies have confirmed that platelets in COVID-19 patients display a hyperactivated phenotype with altered gene expression. In a cohort of 115 COVID-19 patients, featuring both non-severe and severe disease, platelets secreted increased IL-1beta and soluble CD40 ligand compared to healthy controls (46). Furthermore, circulating levels of serotonin and PF4 were increased in patient serum, suggesting platelet degranulation. Common cell markers of platelet activation, including P-selectin and CD63, are also increased in critically ill COVID-19 patients, but not those with mild disease (47). Platelets from critically ill COVID-19 patients also demonstrated increased markers of apoptosis, such as phosphatidylserine externalization and cleaved-caspase 9, which correlate with thromboembolic events (48). In addition to platelet activation, there is evidence of unique transcriptome changes that occur in platelets from COVID-19 patients. Using RNA-seq analysis on platelets from 10 COVID-19 patients, Manne et al. demonstrated significant upregulation of genes involved in antigen presentation (49). Platelets are thus significantly altered to a more active phenotype in COVID-19, particularly in critically ill patients, and may contribute to clinical presentation.

One mechanism by which platelets may contribute to COVID-19 presentation is through neutrophil recruitment and aggregation. As previously noted, platelet P-selectin is able to bind neutrophil ligands to induce rolling and aggregation at sites of activation (50). This interaction can lead to prothrombotic platelet-neutrophil aggregates as well as the formation of neutrophil extracellular traps. For example, plasma from hospitalized COVID-19 patients demonstrates increased circulating platelet-neutrophil aggregates on flow cytometry compared to healthy controls (51). Furthermore, autopsies in COVID-19 patients confirm the presence of microvascular thrombi consisting of neutrophil extracellular traps and platelets (52, 53). These platelet-neutrophil interactions are more prominent in critically ill COVID-19 patients, where there is evidence of a hyperactivated platelet phenotype (52, 54). Therefore, hyperactivated platelets in COVID-19 also contribute to neutrophil activation, which fuels the thrombo-inflammatory milieu.

It is still unclear as to what triggers such drastic platelet changes in critically ill patients. Some have hypothesized that SARS-CoV-2 directly interacts with platelets to mediate these observed effects. Evidence for this is supported by the presence of viral RNA in platelets of infected individuals, although this is only seen in up to 24% of patients (46, 49, 55). However, aside from a single study (55), multiple studies have failed to demonstrate ACE2 expression on the platelet surface or evidence of ACE2 RNA in platelets (46, 49). The cause of this discrepancy is unclear and may be related to different techniques for platelet isolation (56). Regardless, SARS-CoV-2 RNA has been consistently found within platelets and thus suggests that ACE2-independent mechanisms of entry exist. Interestingly, when critically ill COVID-19 patient plasma is incubated with platelets from healthy volunteers, there is a similar increase in platelet activation markers (P-selectin, CD63) (47). While circulating virus may account for this change as well, other soluble mediators should be considered.

## The “COVID Complex” – Immune Complex Mediated Platelet Activation

Immune complexes are one potential circulating factor that could contribute to platelet activation in COVID-19. As previously mentioned, immune complexes activate platelets through the FcγRIIa and may be formed from antibodies against self or exogenous antigens. Viral illnesses are well documented to produce antibodies against self-antigens, such as antiphospholipid antibodies, through a process called molecular mimicry. Early reports in COVID-19 patients highlighted the presence of these antibodies in association with thrombosis, including anti-beta-2 glycoprotein and non-specific inhibitor (57–59). Injection of the serum IgG fraction from these patients into mice resulted in significantly increased thrombus formation compared to controls (59). However, this thrombus formation was also seen with COVID-19 patient serum that had low levels of antiphospholipid antibodies. This suggests that antiphospholipid antibodies are not the sole antibodies associated with this prothrombotic state.

Another potential hypothesis is that HIT antibodies are contributing to the IgG-mediated platelet activation seen in COVID-19 patients. This is supported by the observations that COVID-19 and HIT share many clinical similarities; COVID-19 patients are often exposed to heparin in the context of hospitalization; and a high proportion of COVID-19 patients test positive for anti-PF4/heparin antibodies on further testing (60, 61). However, in a cohort of ten critically ill COVID-19 patients with high suspicion of HIT, we found no evidence of platelet-activating HIT antibodies, which has been replicated by others (61, 62). Interestingly, six of these samples were able to activate platelets in the serotonin release assay in the absence of heparin. This activation was inhibited by IV.3, an FcγRIIa inhibitor, thus confirming immune complex mediated platelet activation. Furthermore, all patients with platelet activation also contained anti-Spike IgG antibodies targeting SARS-CoV-2. It is plausible that Spike-specific IgG antibodies bind circulating Spike protein in viremic, critically ill patients to form platelet-activating immune complexes. This mechanism has previously been shown to occur with H1N1 influenza virus whereby influenza antibodies bind to virus to form immune complexes (63). These immune complexes activate platelets through the FcγRIIa and likely contribute to the pulmonary thrombosis seen with H1N1 infection (64). Most recently, one *in vitro* study confirmed that recombinant anti-Spike IgG is able to activate platelets through the FcγRIIa (65). This was determined through *in vitro* thrombus measurement using microfluidic flow chips and confocal microscopy. Thrombus formation only occurred in the presence of Spike protein and an “inflammatory signal” (von Willebrand factor in this study). Interestingly, anti-Spike IgG and Spike protein alone did not lead to significant thrombus formation. How exactly von Willebrand factor interacts to promote platelet activation is unclear but may be through facilitating platelet aggregation. Glycosylation status of anti-Spike IgG was also found to be a significant factor in the ability of these complexes to activate platelets. Therefore, certain anti-Spike IgG activate platelets in the context of COVID-19 infection, but this remains to be validated in the clinical context.

## Vaccine Induced Thrombotic Thrombocytopenia (VITT)

It would be remiss to avoid a discussion of vaccine-induced thrombotic thrombocytopenia (VITT) in the context of platelet activation and COVID-19. Although this platelet activation is not directly related to SARS-CoV-2 viral infection, it has important clinical and public health implications. VITT is a novel, “drug” related disorder attributed to vaccination by adenoviral vector-based SARS-CoV-2 vaccines. This primarily includes the ChAd-Ox1 (produced by AstraZeneca) and Ad26.COV2.S (Johnson and Johnson) vaccines. VITT was first described in eleven patients, predominantly female, who presented with unusual thromboses (cerebral venous sinus thrombosis, splanchnic-vein thrombosis) and thrombocytopenia (66). Patients often present a median of 14 days from vaccination and can be critically ill – estimated mortality is 22% in one cohort of 220 VITT cases (67). This mortality is significantly reduced from initial reports (55%), likely due to a combination of increased recognition and better treatment implementation (66). Given the parallels to HIT, it was hypothesized that similar platelet activating antibodies may be the underlying cause. This proved to be the case, with all patients featuring high titers of anti-PF4/heparin antibodies that were able to activate platelets in functional assays (66). Interestingly, these antibodies did not require the presence of heparin to form immune complexes and thus are able to target PF4 independently (i.e. anti-PF4 antibodies). The binding site for these antibodies is located in the heparin-binding site on PF4, as shown by alanine-scanning mutagenesis, thus allowing them to form tetrameric immune complexes and activate platelets through FcγRIIa (68). This competitive binding to the heparin site likely explains why heparin inhibits VITT platelet activation *in vitro* (unlike in HIT, where heparin facilitates activation; see **Figure 1**). As previously mentioned, anti-PF4/heparin antibodies do not appear to be responsible for the thrombosis and platelet activation seen in COVID-19. In a cohort of 222 COVID-19 patients with thrombosis, only nineteen (8.6%) tested positive for anti-PF4/heparin antibodies (69). None of these were able to activate platelets in the functional platelet assay. Therefore, anti-PF4 antibodies are likely not responsible for the thrombosis seen in COVID-19 patients and do not demonstrate cross reactivity with the Spike protein. This understanding is important for future vaccine development and management of these rare cases.

## Summary

Although the field of COVID-19 thrombosis is in its infancy, there is sufficient evidence to support a major role for platelets in disease pathogenesis. Platelets have been shown to be hyperactivated in critically ill patients and secrete excessive procoagulant molecules. Furthermore, they are able to interact with other immune cells to mediate the immune response. However, this excess inflammation may also contribute to tissue damage and ultimately, mortality. Targeting these pathways in order to dampen the excess immune response may thus present attractive therapeutic targets.

Immune complexes also appear to contribute significantly to these platelet changes in a manner similar to HIT and other immune-complex mediated disorders. The antigen specificity and additional characteristics of these immune complexes remain to be determined but will be crucial to the development of therapeutic targets. Identifying the specific antibodies involved may also allow clinicians to risk stratify patients who are at high risk of severe disease or thrombosis, thus offering the potential for prophylactic anticoagulation.

## Author Contributions

Both SJ and IN contributed equally to this work, including initial manuscript preparation, editing, and final drafting. All authors contributed to the article and approved the submitted version. 

## Funding

Funding was received from the Canadian Institutes of Health Research (CIHR 452655) awarded to IN.

## Conflict of Interest

The authors declare that the research was conducted in the absence of any commercial or financial relationships that could be construed as a potential conflict of interest.

## Publisher’s Note

All claims expressed in this article are solely those of the authors and do not necessarily represent those of their affiliated organizations, or those of the publisher, the editors and the reviewers. Any product that may be evaluated in this article, or claim that may be made by its manufacturer, is not guaranteed or endorsed by the publisher.
